# Supplementary material for: Incidence and risk factors of omicron variant SARS-CoV-2 breakthrough infection among vaccinated and boosted individuals
Source: medRxiv. 2024 Apr 5:2024.04.03.24305293. Preprint. [Version 1] doi: 10.1101/2024.04.03.24305293 (PMC11023664; doi:10.1101/2024.04.03.24305293)
Supplement: Supplement 1 [file NIHPP2024.04.03.24305293v1-supplement-1.pdf]

medRxiv preprint doi: https://doi.org/10.1101/2024.04.03.24305293; this version posted April 5, 2024. The copyright holder for this preprint (which was not certified by peer review) is the author/funder, who has granted medRxiv a license to display the preprint in perpetuity.
It is made available under a CC-BY 4.0 International license .

Supporting Information

S1 Dataset. Vaccine Cohort Dataset.

S2 Supporting Information. Vaccine Cohort Dataset Codebook
